# Feasibility and safety of only DCB approach in consecutive patients undergoing contemporary CTO PCI; the all-comers DCB study

**DOI:** 10.3389/fcvm.2026.1889893

**Published:** 2026-08-05

**Authors:** Sevket Gorgulu, Sefa Sural, Mahmut Uluganyan, Hasim Tuner, Vedat Aslan, Dimitrios Strepkos, Ahmet Akyel, Hasan Ali Barman, Omer Dogan, Turgut Uygun, Cuneyt Kocas, Emmanouil S. Brilakis, Omer Goktekin

**Affiliations:** 1 Cardiology Department, Biruni University Medical School, Istanbul, Türkiye; 2 Cardiology Department, Toros University, Mersin, Turkiye; 3 Cardiology Department, Bezmialem Vakif University, Istanbul, Türkiye; 4 Cardiology Department, Memorial Bahcelievler Hospital, Istanbul, Türkiye; 5 Cardiology Department, Minneapolis Heart Institute and Minneapolis Heart Institute Foundation, Abbott Northwestern Hospital, Minneapolis, MN, United States; 6 Cardiology Department, Istanbul University, Cerrahpasa Institute of Cardiology, Istanbul, Türkiye; 7 Cardiology Department, Konya City Hospital, Istanbul, Türkiye

**Keywords:** chronic total occlusion, CTO PCI, DCB-only strategy, drug-coated balloon, percutaneous coronary intervention, restenosis, target vessel failure

## Abstract

**Background:**

Evidence regarding an “only drug-coated balloon (DCB)” strategy in chronic total occlusion (CTO) percutaneous coronary intervention (PCI) remains limited, particularly in complex all-comer populations. We aimed to evaluate the feasibility, safety, and mid-term outcomes of an only DCB strategy in consecutive patients undergoing contemporary CTO PCI.

**Methods:**

This multicenter observational study included consecutive CTO PCI procedures performed between January 2024 and April 2026 at three high-volume CTO centers. All procedures were initially attempted using an intention-to-treat “only DCB” strategy. The primary endpoint was major adverse cardiac and cerebrovascular events (MACCE), defined as cardiac death, target lesion revascularization (TLR), target vessel myocardial infarction, or stroke.

**Results:**

A total of 457 CTO PCI procedures were evaluated. After excluding unsuccessful guidewire crossing procedures, 423 lesions underwent lesion preparation using the only DCB protocol. Final treatment with an “only DCB” strategy was achieved in 248 lesions, corresponding to a feasibility rate of 58.62%. Mean J-CTO score was 1.97 ± 1.04. Antegrade wiring was used in 95.2% of procedures, whereas retrograde wiring was used in 16.9%. Mean follow-up duration was 231.1 ± 130.3 days. MACCE occurred in 28 patients (13.9%), and target vessel failure in 27 (13.4%). Follow-up mortality occurred in one patient (0.5%), and no patient had a stroke. Patients with angiographic restenosis ≥70% had smaller final minimum lumen diameter (1.69 ± 0.64 mm vs. 2.23 ± 0.80 mm; *p* = 0.001). Among patients with angiographic follow-up, late lumen gain was observed in 55.5% of lesions, whereas lumen gain>0.2 mm was observed in 45.4%, supporting positive vessel remodeling after DCB treatment.

**Conclusions:**

In an all-comer CTO PCI population, an only DCB strategy appears feasible in more than half of patients, with acceptable mid-term clinical and angiographic outcomes.

## Graphical Abstract

**Figure:**
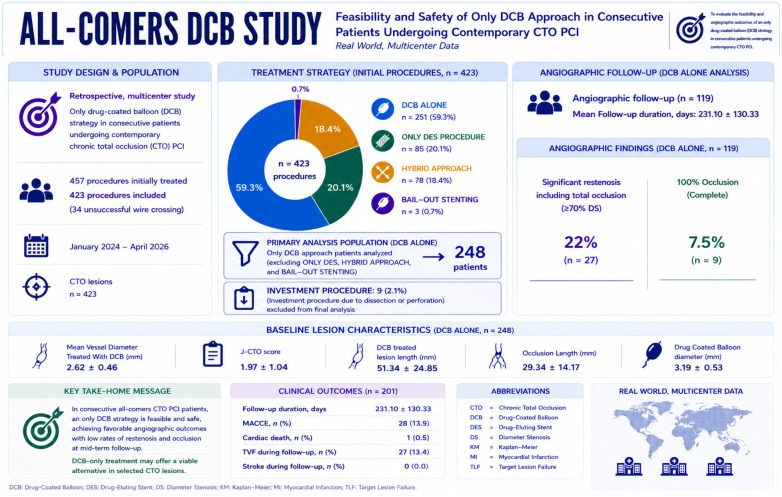

## 1 Introduction

Chronic total occlusion (CTO) percutaneous coronary intervention (PCI) can be challenging to perform (1–4). Drug-eluting stent (DES) implantation significantly reduces restenosis in CTO PCI compared with balloon angioplasty and bare metal stent implantation, but still carries risks of restenosis and stent thrombosis, and does not allow for positive vessel remodeling (5). Moreover, prolonged dual antiplatelet therapy (DAPT) is associated with increased risk of bleeding (6, 7). Drug-coated balloons (DCBs) have emerged as an alternative to DES in this setting, either alone or as an adjunct to DES, with promising results and increasing use over time (8–11). Therefore, DCBs are often used in patients with lower complexity, small-vessels, or distal lesions (12–16).

Since 2024 three centers adopted a “DCB first” approach to CTO PCI. Herein, we report the outcomes of patients treated at those centers during this period of time.

## 2 Materials and methods

### 2.1 Study population

**Figure 1:**
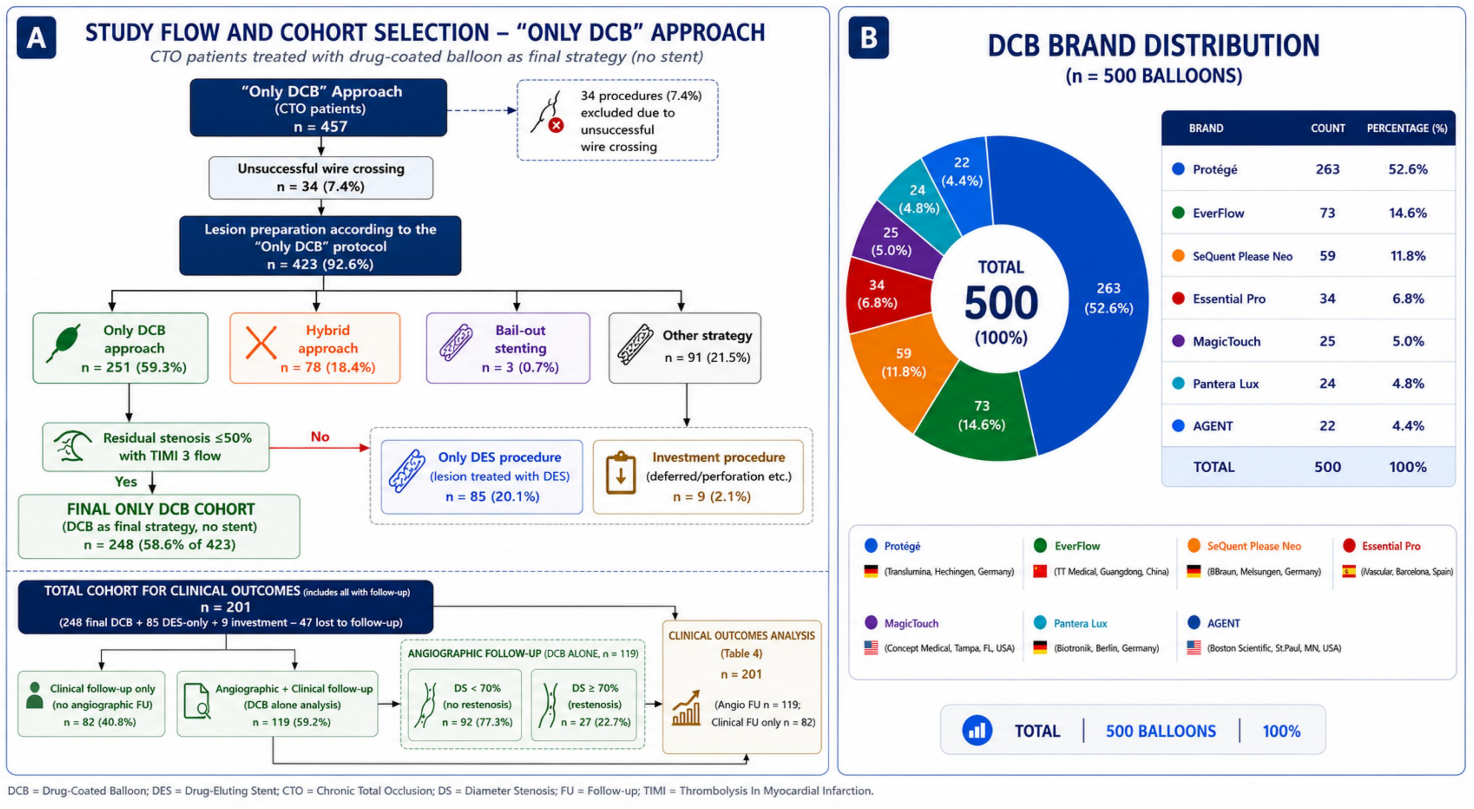
Study flowchart and DCB usage distribution.

As shown in Figure 1A, we analyzed the clinical and angiographic characteristics and procedural outcomes of consecutive CTO PCIs performed between January 2024,1 and April 2026,1 at 3 centers: Biruni University School of Medicine, Istanbul; Memorial Bahçelievler Hospital, Istanbul; and Bezmialem University, Istanbul. The Data collection was recorded in a dedicated online database [PROGRESS-CTO (Prospective Global Registry for the Study of Chronic Total Occlusion Intervention); Clinicaltrials.gov identifier: NCT02061436]. The study data were collected and managed using REDCap (Research Electronic Data Capture) hosted at the Minneapolis Heart Institute Foundation (17). The data in the study were retrospectively analyzed following approval from the secondary ethics committee. The study was approved by the Mersin University Ethics Committee with decision number 2026/297.

Every patient undergoing CTO PCI entered the cath lab with the intention of an “only DCB” approach. Unsuccessful guidewire crossing procedures and DES implantation procedures covering any CTO segment with DCB (hybrid approach) or without DCB were excluded from the study. The use of DES ≥5 mm outside of the occlusion site was not deemed as an exclusion criterion (15).

### 2.2 Definitions and endpoints

A CTO was defined as a coronary total occlusion with TIMI (Thrombolysis in Myocardial Infarction) flow grade 0 for more than 3 months. Because the nomenclature suggested by the CTO-ARC Consensus Recommendations ideally summarizes all CTO crossing techniques, the definition of a lesion-crossing strategy was accordingly considered (18).

Technical success was defined as a final residual stenosis of ≤50% on quantitative coronary angiography (QCA) and TIMI flow grade 3 after DCB treatment (12). Procedural success was defined as technical success without any in-hospital major adverse cardiovascular event (MACE). In-hospital MACE, defined as death, myocardial infarction (MI), repeat target vessel revascularization with PCI or coronary artery bypass graft (CABG) surgery, tamponade requiring either pericardiocentesis or surgery, and stroke. Stroke was defined as an acute neurological event lasting ≥24 h. Type 4a MI was the definition of MI (19).

All patients treated with the “Only DCB” strategy underwent scheduled clinical follow-up at 1, 3, 6, and 12 months. Patients were instructed to promptly report any typical or atypical cardiac symptoms during follow-up. Repeat coronary angiography (CAG) was performed selectively in patients with a clinical suspicion of ischemia or target vessel failure — including recurrent chest pain, angina-equivalent symptoms, an acute coronary syndrome (ACS) presentation, or evidence of ischemia on non-invasive testing — after additional informed consent. Routine follow-up CAG was not performed in asymptomatic patients. Patients without clinical or angiographic evidence of adverse cardiovascular events during follow-up were considered free of major adverse cardiac and cerebrovascular events (MACCE).

Quantitative coronary angiographic (QCA) analysis of the region of interest was performed as previously described (20). Minimal lumen diameter (MLD), acute recoil, and late lumen loss (LLL) were assessed according to the consensus definitions of the Drug-Coated Balloon Academic Research Consortium (20).

Target vessel failure (TVF) was defined as angiographically confirmed restenosis of the DCB-treated vessel with diameter stenosis ≥70%, or complete re-occlusion, detected at clinically indicated follow-up CAG. Clinically driven target lesion or target vessel revascularization (TLR/TVR) — i.e., repeat PCI or CABG of the treated lesion segment (TLR) or the DCB-treated vessel (TVR) — represented the subgroup of TVF that required revascularization, since in this cohort all repeat revascularizations were preceded by angiographic confirmation of ≥70% restenosis or re-occlusion. Angiographic TVF without clinically driven revascularization was reported separately as angiographic-only TVF.

The primary outcome of the study was the incidence of MACCE, defined as a composite of cardiac death, target-vessel myocardial infarction (TVMI), stroke, and TVF during follow-up. When more than one component occurred in the same patient, the first event was used for time-to-event analyses.

Secondary outcomes included angiographic-only TVF, clinically driven TLR/TVR, late lumen loss (LLL), and symptomatic status during follow-up. Symptomatic status — including angina, dyspnea, and fatigue — was categorized as symptom improvement, no change, or worsening.

The J-CTO (Multicenter CTO registry in Japan), the PROGRESS CTO (Prospective Global Registry for the Study of Chronic Total Occlusion Intervention) and the PROGRESS CTO complication scores were calculated as previously described (21–23).

Bail out stenting is defined as: DES deployment after DCB usage due to various reasons as previously described (20).

### 2.3 Lesion preparation and DCB application

Lesion preparation was done with scoring and cutting balloon, if available, if not, at least 20 mm long NC balloons were preferred. After lesion preparation, the D**C**B brands specified in Figure 1B were used. The inflation time of DCBs was per the manufacturer's recommendation, and DCBs at least 5 mm longer than the actual lesion were selected to avoid longitudinal geographic miss. In cases where >1 DCB was required, they were overlapped by at least 1 mm. After appropriate lesion preparation, lesions with flow-limiting dissections, acute recoil with residual stenosis >50%, or a vessel hematoma with luminal collapse were converted to stenting. The same criteria were applied for bailout stenting. Lesions without flow deterioration or significant acute recoil were not stented, even if the dissection was type C or higher. Dissections were classified according to the National Heart, Lung, and Blood Institute classification system for intimal tears, consisting of Types A through F (24).

### 2.4 Statistical analysis

Continuous variables were assessed for distributional characteristics and are presented as mean ± standard deviation or median (interquartile range), as appropriate. Categorical variables are summarized as counts and percentages. Comparisons between groups were performed according to variable type and distribution. Continuous variables were compared using the independent-samples t-test for normally distributed data and the Mann–Whitney U test for non-normally distributed data. Categorical variables were compared using the chi-square test or Fisher's exact test, as appropriate. All tests were two-sided, and a *p*-value <0.05 was considered statistically significant.

Time-to-event analyses were performed using the Kaplan–Meier method. Freedom from MACCE was estimated from the index procedure to the first documented MACCE event. Patients without events were censored at the last available clinical or angiographic follow-up. Where appropriate, survival curves were compared using the log-rank test.

To identify independent predictors of MACCE in the Only DCB cohort, a multivariable Cox proportional hazards regression model was constructed. Covariates included clinically relevant baseline and procedural characteristics (age, sex, diabetes mellitus, J-CTO score, prior CABG, and left ventricular ejection fraction). Results are reported as hazard ratios (HRs) with 95% confidence intervals (CIs). The proportional hazards assumption was assessed using standard diagnostic methods. Given the limited number of events, multivariable results should be interpreted as exploratory. Patients with missing data for a given variable were excluded from the corresponding analysis. No imputation was performed. Time-to-event data were expressed in months. All statistical analyses were performed with R Statistical Software, version 4.4 (R Foundation for Statistical Computing, Vienna, Austria).

## 3 Results

### 3.1 Study flow and patient population

Between January 2024 and April 2026, a total of 457 CTO PCI procedures were initially planned using a drug-coated balloon (DCB)-only strategy. Of these, 34 procedures (7.4%) were excluded due to unsuccessful wire crossing, leaving 423 procedures in which lesion preparation under the “only DCB” protocol was successfully performed. The initial treatment strategy consisted of DCB-only in 251 patients (59.3%), a hybrid approach in 78 patients (18.4%), and other strategies in 91 patients (21.5%).

Among the 251 patients treated with a DCB-only strategy, 248 (58.6%) achieved residual stenosis ≤50% with TIMI 3 flow, constituting the final DCB-only cohort; 3 ptients underwent bailout stenting (0.7%). Follow-up data were available in 201 patients, who were included in the clinical outcomes analysis. Of these, 119 patients (59.2%) underwent both angiographic and clinical follow-up, whereas 82 patients (40.8%) had clinical follow-up only (Figure 1A and Supplementary Figure S1).

A total of 500 DCBs were used during the study period. Protégé was the most frequently used DCB brand (52.6%), followed by EverFlow (14.6%) and SeQuent Please Neo (11.8%) (Figure 1B).

### 3.2 Baseline clinical characteristics

The mean age of the study population was 60.7 ± 9.7 years, and 85.1% of patients were male. Diabetes mellitus was present in 49.2% of patients, hypertension in 75.4%, dyslipidemia in 55.2%, and active smoking in 75.4%. Previous myocardial infarction was reported in 42.3% of patients, prior PCI in 60.5%, and prior CABG in 9.7%. Mean left ventricular ejection fraction (LVEF) was 50.0 ± 10.4% (Table 1).

**Table 1:** Baseline clinical characteristics.

Parameter	Overall cohort (*n* = 248)	Angiographic follow-up DS <70% (*n* = 92)	Angiographic follow-up DS ≥70% (*n* = 27)	Clinical follow-up only (*n* = 82)	*p*-value
Age, years	60.7 ± 9.7	59.5 ± 10.1	63.1 ± 9.7	61.1 ± 9.9	0.100
Male sex, *n* (%)	211 (85.1)	84 (91.3)	21 (77.8)	69 (84.1)	0.084
Height, cm	169.9 ± 9.1	171.8 ± 8.7	168.4 ± 10.3	169.4 ± 8.7	0.127
Weight, kg	83.7 ± 15.1	83.4 ± 15.5	83.9 ± 13.7	84.8 ± 12.9	0.872
BMI, kg/m^2^	29.1 ± 5.4	28.2 ± 5.0	29.6 ± 4.5	29.7 ± 5.1	0.173
*Medical history*					
Diabetes mellitus, *n* (%)	122 (49.2)	44 (47.8)	16 (59.3)	42 (51.2)	0.382
Hypertension, *n* (%)	187 (75.4)	65 (70.7)	22 (81.5)	64 (78.0)	0.329
Hypercholesterolemia, *n* (%)	156 (62.9)	62 (67.4)	16 (59.3)	47 (57.3)	0.492
Current smoker, *n* (%)	187 (75.4)	72 (78.3)	19 (70.4)	61 (74.4)	0.442
Family history of CAD, *n* (%)	96 (38.7)	32 (34.8)	7 (25.9)	34 (41.5)	0.487
History of heart failure, *n* (%)	33 (13.3)	10 (10.9)	3 (11.1)	7 (8.5)	1.000
Peripheral arterial disease, *n* (%)	11 (4.4)	5 (5.4)	2 (7.4)	2 (2.4)	0.656
Previous MI, *n* (%)	105 (42.3)	36 (39.1)	11 (40.7)	37 (45.1)	1.000
Previous PCI, *n* (%)	150 (60.5)	44 (47.8)	13 (48.1)	60 (73.2)	1.000
Previous CABG, *n* (%)	24 (9.7)	3 (3.3)	5 (18.5)	7 (8.5)	**0**.**015**
Previous TIA or stroke, *n* (%)	5 (2.0)	2 (2.2)	1 (3.7)	1 (1.2)	0.541
Hgb, g/dL	13.6 ± 1.8	13.7 ± 2.1	13.1 ± 1.4	13.7 ± 1.5	0.089
Creatinine, mg/dL	1.0 ± 0.3	1.0 ± 0.2	1.0 ± 0.4	1.0 ± 0.3	1.000
LVEF, %	50.0 ± 10.4	51.0 ± 9.4	48.5 ± 8.3	51.6 ± 10.5	0.189
*Clinical presentation*					
Stable angina, *n* (%)	161 (64.9)	64 (69.6)	18 (66.7)	52 (63.4)	0.815
Unstable angina, *n* (%)	2 (0.8)	0 (0.0)	1 (3.7)	1 (1.2)	0.227
No symptoms, *n* (%)	67 (27.0)	22 (23.9)	7 (25.9)	25 (30.5)	0.804

Data are presented as mean ± SD or n (%). Continuous variables were compared using the independent-samples t-test or Mann–Whitney U test, and categorical variables using the chi-square test or Fisher's exact test, as appropriate. A two-sided *p*-value <0.05 was considered statistically significant.

Subgroup columns include patients with available follow-up data, whereas the overall cohort includes all enrolled patients.

BMI, body mass index; CABG, coronary artery bypass grafting; CAD, coronary artery disease; DCB, drug-coated balloon; DS, diameter stenosis; FU, follow-up; Hgb, hemoglobin; LVEF, left ventricular ejection fraction; MI, myocardial infarction; PCI, percutaneous coronary intervention; TIA, transient ischemic attack.

Clinical baseline characteristics were generally comparable between patients with angiographic restenosis (DS ≥70%) and those without restenosis. However, a history of CABG was more frequent among patients who developed restenosis (18.5% vs. 3.3%, *p* = 0.015) (Table 1).

### 3.3 Lesion and angiographic characteristics

As shown in Table 2, the mean J-CTO score was 1.97 ± 1.04. CTO lesions were most commonly located in the RCA (46.0%) and LAD (32.7%). Mean DCB-treated lesion length was 51.3 ± 24.9 mm, whereas mean occlusion length was 29.3 ± 14.2 mm.

**Table 2:** Lesion and angiographic characteristics.

Variables	Overall (*n* = 248)	Angiographic follow-up DS <70% (*n* = 92)	Angiographic follow-up DS ≥70% (*n* = 27)	*P* value
J-CTO score	1.97 ± 1.04	1.82 ± 1.00	2.05 ± 1.00	0.299
Progress-CTO score	1.01 ± 0.89	0.87 ± 0.84	0.77 ± 0.69	0.532
Progress-CTO complication score	2.62 ± 1.71	2.35 ± 1.54	2.78 ± 1.85	0.277
Proximal reference vessel diameter, mm	2.85 ± 0.53	2.81 ± 0.50	3.08 ± 0.62	**0**.**045**
Distal reference vessel diameter, mm	2.34 ± 0.56	2.33 ± 0.55	2.37 ± 0.66	0.814
Mean vessel diameter treated with DCB, mm	2.62 ± 0.46	2.61 ± 0.40	2.67 ± 0.50	0.634
DCB-treated lesion length, mm	51.34 ± 24.85	49.37 ± 24.48	44.46 ± 17.79	0.262
Occlusion length, mm	29.34 ± 14.17	24.92 ± 9.52	32.07 ± 21.87	0.109
Minimum lumen diameter at final PCI, mm	1.74 ± 0.48	1.77 ± 0.47	1.60 ± 0.52	0.200
Minimum lumen diameter at follow-up, mm	1.82 ± 0.92	2.09 ± 0.70	0.40 ± 0.53	**<0**.**001**
Late lumen loss	−0.07 ± 0.84	−0.32 ± 0.62	1.20 ± 0.64	**<0**.**001**
Diameter stenosis at final PCI, %	23.84 ± 18.41	16.59 ± 18.77	25.67 ± 20.59	**0**.**047**
Diameter stenosis at follow-up, %	38.37 ± 30.09	25.68 ± 15.40	91.99 ± 12.24	**<0**.**001**
Drug-coated balloon diameter, mm	3.19 ± 0.53	3.22 ± 0.50	3.28 ± 0.58	0.672
Drug-coated balloon total length, mm	44.57 ± 21.43	49.37 ± 24.48	44.46 ± 17.79	0.262
Number of balloons per lesion	1.56 ± 0.71	1.55 ± 0.70	1.73 ± 0.78	0.291
In-stent CTO, *n* (%)	46 (18.5)	12 (13.0)	5 (18.5)	0.534
LAD CTO, *n* (%)	81 (32.7)	42 (45.7)	9 (33.3)	0.278
CX CTO, *n* (%)	50 (20.2)	13 (14.1)	6 (22.2)	0.371
RCA CTO, *n* (%)	114 (46.0)	36 (39.1)	12 (44.4)	0.660
Moderate or severe calcification, *n* (%)	99 (39.9)	35 (38.0)	9 (33.3)	0.821
*Dissection classification*	***n*** **=** **147**	***n*** **=** **53**	***n*** **=** **14**	
Type A, *n* (%)	48 (32.7)	22 (41.5)	5 (35.7)	0.767
Type B, *n* (%)	63 (42.9)	21 (39.6)	8 (57.1)	0.364
Type C, *n* (%)	18 (12.2)	6 (11.3)	1 (7.1)	1.000
Type D, *n* (%)	17 (11.6)	3 (5.7)	0 (0.0)	1.000
Type E, *n* (%)	1 (0.7)	1 (1.9)	0 (0.0)	1.000
Type F, *n* (%)	0 (0.0)	0 (0.0)	0 (0.0)	1.000

Data are presented as mean ± SD or n (%). Bold values indicate statistical significance (*P* < 0.05).

Follow-up angiographic variables were analyzed only in patients with available follow-up angiography.

CTO, chronic total occlusion; DCB, drug-coated balloon; DS, diameter stenosis; FU, follow-up; J-CTO, Japanese CTO score; LAD, left anterior descending artery; Cx, Circumflex artery; PCI, percutaneous coronary intervention; RCA, right coronary artery.

Patients who developed restenosis during angiographic follow-up had significantly larger proximal reference vessel diameter compared with patients without restenosis (3.08 ± 0.62 mm vs. 2.81 ± 0.50 mm, *p* = 0.045). Final post-procedural minimum lumen diameter was significantly lower in the restenosis group (1.69 ± 0.64 mm vs. 2.23 ± 0.80 mm, *p* = 0.001). Similarly, follow-up minimum lumen diameter remained significantly lower in the restenosis group (1.60 ± 0.52 mm vs. 2.64 ± 0.95 mm, *p* = 0.016) (Table 2).

Late lumen loss was substantially greater in patients with DS ≥70% restenosis (1.20 ± 0.64 mm vs. −0.32 ± 0.62 mm, *p* < 0.001). Final post-PCI diameter stenosis was also significantly higher in the restenosis group (25.7 ± 20.6% vs. 16.6 ± 18.8%, *p* = 0.047) (Table 2).

Scatter plot analysis demonstrated a weak correlation between post-procedural and follow-up minimum lumen diameter measurements (Pearson r = 0.07) (Supplementary Figure S2).

### 3.4 Procedural characteristics

Antegrade wiring was the predominant crossing strategy and was used in 95.2% of procedures. Retrograde crossing was performed in 16.9% of cases. Mean procedural duration was 78.7 ± 44.3 min, mean fluoroscopy time was 35.6 ± 23.4 min, and mean contrast volume was 218 ± 109 mL (Table 3).

**Table 3:** Procedural characteristics.

Variables	Overall (*n* = 248)	Angiographic follow-up DS <70% (*n* = 92)	Angiographic follow-up DS ≥70% (*n* = 27)	*P* value
*Crossing strategy*				
Antegrade wiring, *n* (%)	236 (95.2)	90 (97.8)	24 (88.9)	0.076
Antegrade dissection and re-entry, *n* (%)	9 (3.6)	2 (2.2)	0 (0.0)	1.000
Retrograde wiring, *n* (%)	42 (16.9)	12 (13.0)	4 (14.8)	0.757
*Lesion characteristics*				
Balloon uncrossable CTO lesion, *n* (%)	9 (3.6)	3 (3.3)	1 (3.7)	1.000
Balloon undilatable CTO lesion, *n* (%)	9 (3.6)	8 (8.7)	0 (0.0)	0.196
*Procedural outcomes*				
Procedure time, min	78.67 ± 44.25	70.69 ± 38.76	73.00 ± 32.88	0.767
Contrast volume, mL	218.21 ± 109.30	223.43 ± 120.30	231.43 ± 80.70	0.744
Fluoroscopy time, min	35.60 ± 23.38	33.63 ± 20.87	34.51 ± 17.50	0.854
Patient air kerma radiation dose, Gy	3.23 ± 2.08	3.60 ± 2.41	3.48 ± 2.10	0.835
Lowest procedural ACT, s	303.75 ± 103.00	338.70 ± 133.88	283.54 ± 86.25	0.143
*Lesion preparation*				
Cutting balloon, *n* (%)	50 (20.2)	16 (17.4)	3 (11.1)	0.559
Scoring balloon, *n* (%)	193 (77.8)	74 (80.4)	16 (59.3)	**0**.**039**
NC balloon, *n* (%)	46 (18.5)	16 (17.4)	8 (29.6)	0.179
Rotablation, *n* (%)	4 (1.6)	3 (3.3)	1 (3.7)	1.000
IVUS used, *n* (%)	56 (22.6)	22 (23.9)	4 (14.8)	0.430

Data are presented as mean ± SD or n (%). Continuous variables were compared using the independent-samples t-test or Mann–Whitney U test, and categorical variables using the chi-square test or Fisher's exact test, as appropriate. Bold values indicate statistical significance (*P* < 0.05).

Follow-up angiographic variables were analyzed only in patients with available follow-up angiography.

Crossing strategies were not mutually exclusive, and multiple crossing strategies could be applied during a single CTO procedure; therefore, percentages may exceed 100%.

ACT, activated clotting time; CTO, chronic total occlusion; DCB, drug-coated balloon; DS, diameter stenosis; IVUS, intravascular ultrasound; NC, non-compliant.

Scoring balloon use during lesion preparation was significantly more common in patients without restenosis compared with those who developed restenosis (80.4% vs. 59.3%, *p* = 0.039). No other procedural variables significantly differed between groups (Table 3).

### 3.5 Clinical and angiographic follow-up outcomes

Mean follow-up duration was 231.1 ± 130.3 days. The overall MACCE rate was 13.9%. Cardiac death occurred in only 1 patient (0.5%), and no stroke events were observed during follow-up (Table 4).

**Table 4:** Clinical follow-up outcomes.

Variables	Overall (*n* = 201)	Angiographic follow-up (*n* = 119)	Clinical follow-up only (*n* = 82)
Follow-up duration, days	231.10 ± 130.33	203.49 ± 119.30	265.44 ± 135.91
MACCE, *n* (%)	28 (13.9)	27 (22.7)	1 (1.2)
Cardiac death, *n* (%)	1 (0.5)	0 (0.0)	1 (1.2)
TVF during follow-up, *n* (%)	27 (13.4)	27 (22.7)	0 (0.0)
Stroke during follow-up, *n* (%)	0 (0.0)	0 (0.0)	0 (0.0)
All-cause mortality, n (%)	3 (1.4)	0 (0.0)	3 (3.7)
*Angina after CTO PCI*			
Improved, *n* (%)	144 (77.8)	87 (78.4)	57 (77.0)
Same, *n* (%)	33 (17.8)	17 (15.3)	16 (21.6)
Worsened, *n* (%)	8 (4.3)	7 (6.3)	1 (1.4)
*Dyspnea after CTO PCI*			
Improved, *n* (%)	123 (75.9)	63 (70.8)	60 (82.2)
Same, *n* (%)	33 (20.4)	20 (22.5)	13 (17.8)
Worsened, *n* (%)	6 (3.7)	6 (6.7)	0 (0.0)
*Fatigue after CTO PCI*			
Improved, *n* (%)	132 (75.9)	75 (74.3)	57 (78.1)
Same, *n* (%)	39 (22.4)	23 (22.8)	16 (21.9)
Worsened, *n* (%)	3 (1.7)	3 (3.0)	0 (0.0)

Data are presented as mean ± SD or n (%). Continuous variables were summarized descriptively. Categorical variables are presented as counts and percentages.

Percentages for symptom outcomes were calculated among patients with available symptom follow-up data.

CTO, chronic total occlusion; MACCE, major adverse cardiac and cerebrovascular events; PCI, percutaneous coronary intervention; TVF, target vessel failure.

Target vessel failure (TVF) occurred in 13.4% of patients. Among the 119 patients with angiographic follow-up, 27 patients (22.7%) developed significant restenosis (DS ≥70%). Complete re-occlusion occurred in 7.5% (*n* = 9) of lesions.

Among the 119 patients with clinically-indicated angiographic follow-up, paired post-DCB and follow-up minimum lumen diameter measurements were available in 110 patients. Late lumen gain, defined as any increase in minimum lumen diameter at follow-up, was observed in 66 patients, corresponding to 55.5% of the angiographic follow-up cohort and 60.0% of evaluable patients. A lumen gain>0.2 mm was observed in 54 patients, corresponding to 45.4% of the angiographic follow-up cohort and 49.1% of evaluable patients. Overall, late lumen enlargement was observed in a substantial proportion of lesions during angiographic follow-up, supporting positive vessel remodeling after DCB treatment.

Symptomatic improvement was observed in most patients. Angina symptoms improved in 77.8% of patients, dyspnea improved in 75.9%, and fatigue improved in 75.9% (Table 4).

**Figure 2:**
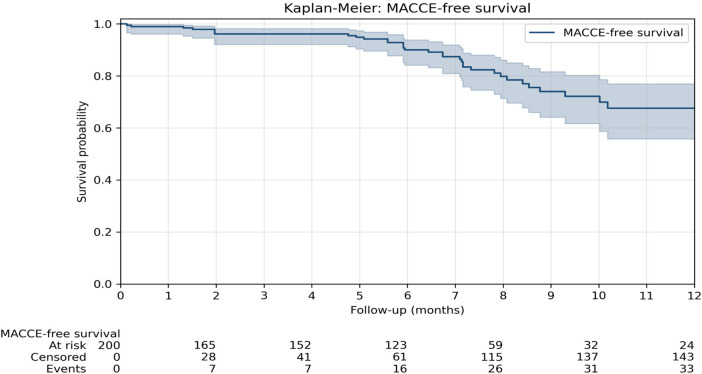
Kaplan meier: MACCE-free survival.

Kaplan–Meier analysis demonstrated preservation of high MACCE-free survival throughout the 12-month follow-up period (Figure 2).

### 3.6 Multivariable Cox regression analysis

Multivariable Cox regression analysis demonstrated that age, male sex, diabetes mellitus, J-CTO score, prior CABG history, and LVEF were not independent predictors of MACCE (Supplementary Figure S3). The hazard ratio for diabetes mellitus was 1.45 (95% CI: 0.70–3.01; *p* = 0.313), while the hazard ratio for J-CTO score was 1.12 (95% CI: 0.76–1.66; *p* = 0.558). None of the evaluated covariates reached statistical significance.

## 4 Discussion

Our study indicates that a DCB Only approach is feasible in 58.6% of an all-comers population and safe, with an acceptable MACCE rate at an intermediate follow-up period.

During the median 8 months of follow-up, the MACCE rate was 13.9%, mainly driven by TVF (13.4%). A meta-analysis (24) including 5 DCB CTO studies (12–14, 25, 26), indicated a 17.2% TVF, which was slightly higher than ours. This difference might be attributable to a longer follow-up period, which was more than 2 years in the two studies (12, 25), and comprised more than half of the study population. A recent study by Opolski et al. (15) reported a 14.4% event rate at 6-month follow-up, which was very similar to our results. The difference in MACE across studies might be dependent on follow-up duration.

The mean CTO difficulty in our cohort was slightly higher (J-CTO 1.97 ± 1.04) than prior DCB only reports, which also showed intermediate J-CTO difficulty scores (J-CTO 1.4 ± 0.6) (12), (J-CTO 1.7 ± 0.9) (14), (J-CTO 1.5 ± 1.1) (15). This discrepancy is not surprising as our cohort consisted of all comers rather than lower complexity patients. Not only was the J-CTO score greater than in prior reports, but our mean DCB diameter (3.19 ± 0.53 mm) was also greater than that reported (ranging from 2.55 to 2.78 mm) (12–15). After DCB use, the minimum lumen diameter was greater in our population (1.96 mm ± 0.68 mm) than in similar studies, which range between 1.6 mm and 1.69 mm (12, 13, 15). Having similar outcomes in larger arteries treated with DCB seems hypothesis-generating and not confining DCB usage to only small vessels, as the DCB consortium has recommended (20).

Among 58.6% of our study population, with a median angiographic follow-up of 8 months, TVF occurred in 22.4% of CTO patients. The binary restenosis rate ranges from 11.5% to 20.8% in “only-DCB” reports (12–14). Opolski et al. (15) reported a 24.2% restenosis rate and attributed this relatively higher rate to lower usage of modified balloons. Our usage rate of scoring/cutting balloons was 90% in the angiographic follow-up group, with less restenosis (Table 3), whereas it was only 18% in the Opolski et al. (15) manuscript.

**Figure 3:**
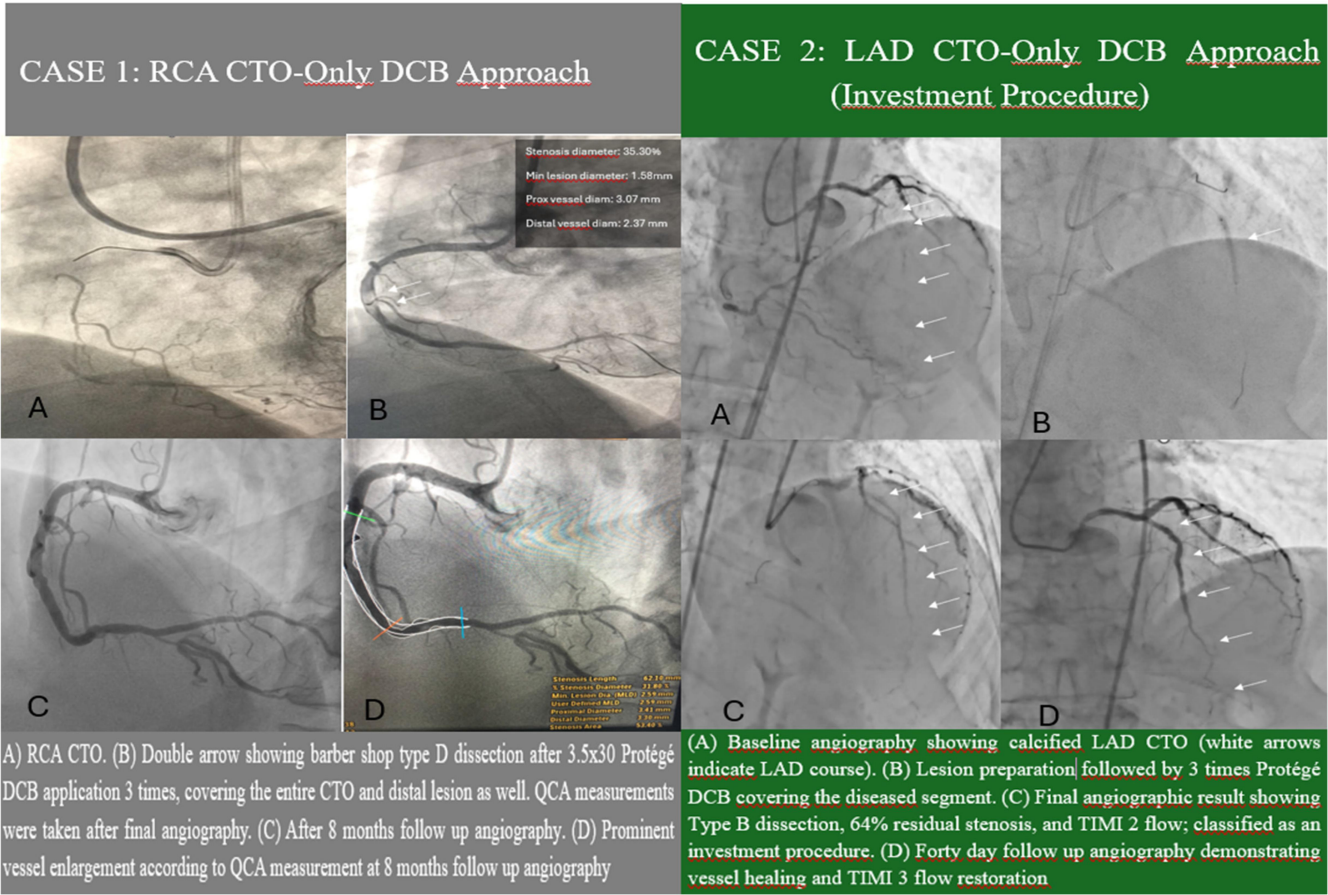
Case examples after DCB application.

What is optimal preparation for DCB only CTO PCI?. The DCB consensus document (27) recommends: a fully inflated balloon of the correct size for the vessel; residual stenosis <30%; TIMI flow grade 3; and the absence of a flow-limiting dissection. This document also states that any type C or higher dissection should be treated with a stent. This might not be the case after CTO PCI, as we have evidence of dissection healing after DCB use (28). Furthermore, it is not standard practice among CTO operators to stent type C, D, or even type F dissections with only-DCB CTO PCI (12, 14, 15). 24.5% of our cases had type C or greater dissections and showed excellent healing on follow-up angiography (Figure 3, Case 1). As CTO operators, we are not afraid of dissections. The worst-case scenario is in-hospital or post-discharge occlusion that was already present before the procedure. The present collateral network will take over and prevent any adverse event. In our series, among 24.5% of cases with type C-E dissections, only 1 patient (7.1%) with a type C dissection had restenosis of ≥70% during angiographic follow-up. The application of DCB in dissected planes may improve outcomes by minimizing aberrant tissue proliferation while safely preserving antegrade flow through the fenestrations (29). Our aim was to achieve TIMI 3 flow before DCB application, but TIMI 2 flow may be sufficient in CTO PCI (15, 17). We sometimes leave patients with TIMI 2 flow after DCB treatment, which heals the dissections and promotes positive vessel enlargement (Figure 3, Case 2). Given possible vessel enlargement, plaque regression, and/or healing of dissection flaps (30, 31), a ≤ 50% residual stenosis or a recoil effect, as seen in the old plain balloon era, should be acceptable for further DCB treatment, which was also used in some CTO DCB studies (12, 14, 25).

Late lumen enlargement was consistently observed across the CTO cohort in a meta-analysis (32). Especially with paclitaxel-coated balloons, dissection healing and vessel enlargement are prominent (33). Our cohort consisted solely of Paclitaxel-coated balloons, with Protégé being the most used (Figure 2). Late lumen enlargement was observed in 66 of 119 patients' lesions (55.5%). In 45.4% (n = 54) of patients, there was late lumen enlargement >0.2 mm. This 45.4% rate seems slightly lower than rates reported in the literature, which reported 50%–74% late lumen enlargement during the early follow-up phase (7–12 months) after intervention (34–36). In studies conducted by the Onishi group (37, 38), LLE occurred frequently in small-vessel CTO lesions, which might explain the lower rate of late lumen enlargement in our study (45.4%). As our cohort was all-comers, large vessels were also part of our only DCB approach.

## Limitations

The limitations of our study are as follows: First, this was an observational study with all inherent limitations. Second, an independent quantitative coronary analysis was not conducted for the angiograms. Third, routine prespecified angiographic follow-up was not available in this study. Therefore, the reported restenosis and re-occlusion rate in the angiographic arm might be overestimated, while the overall TVF might underestimate the silent failure occurrence. Fourth, around 20% of our patients were lost to follow-up; this group usually has a higher event rate, which may lead to an underestimation of the true event rate for both clinical and angiographic endpoints. Fifth, another important limitation of the present study relates to the definition of angiographic restenosis. Although the conventional binary restenosis threshold in the DCB literature is ≥50% diameter stenosis, we used a ≥ 70% cutoff to focus on clinically more significant restenotic lesions in this real-world CTO population. While this approach may better reflect clinically relevant vessel failure, it may also underestimate the overall angiographic restenosis rate and limit direct comparison with prior DCB and DCB-CTO studies that use the standard ≥50% definition.

Sixth, there was no clinical event adjudication by an independent committee. Seventh, technique selection was at the operator's discretion. Eighth, including 6 covariates (age, sex, diabetes, J-CTO score, prior CABG, and LVEF) with a limited number of events carries a high risk of statistical overfitting. Therefore, multivariable results should be interpreted as exploratory. Ninth, restenosis and TVF may occur later, around 12–18 months; our 8-month follow-up results should be interpreted within this specific midterm time frame. Finally, all CTO PCIs were performed at high volume, dedicated to 3 centers with highly experienced 3 operators. This should be considered a limitation, as outcomes may differ in less experienced centers, thereby limiting the generalizability of our findings.

## Conclusion

The only DCB approach in CTO PCI seems feasible in a large proportion of all-comer patients. The DCB CTO Trial (NCT07369765), a randomized non-inferiority trial, will provide more robust evidence on the safety of DCB use in CTO PCI.

## Data Availability

The original contributions presented in the study are included in the article/Supplementary Material, further inquiries can be directed to the corresponding author.
